# Knockdown of pseudogene DUXAP8 expression in glioma suppresses tumor cell proliferation

**DOI:** 10.3892/ol.2020.11859

**Published:** 2020-07-10

**Authors:** Xu Zhao, Shuai Hao, Minqing Wang, Deguang Xing, Chengwei Wang

Oncol Lett 17: 3511-3516, 2019; DOI: 10.3892/ol.2019.9994

Subsequently to the publication of the above article, the authors have realized that Fig. 4A contained an error in the selection of the data: Essentially, the image for the si-RNA-1 experiment with the U87 cell line was inadvertently chosen incorrectly.

A revised version of Fig. 4, including the correct data for the si-RNA-1 experiment for the U87 cell line, is shown opposite. Note that this change does not affect the results or the conclusions reported in this paper, and all the authors agree to this correction. The authors thank the Editor for allowing them the opportunity to publish this corrigendum, and apologize to the readership of the Journal for any inconvenience caused.

## Figures and Tables

**Figure 4.**
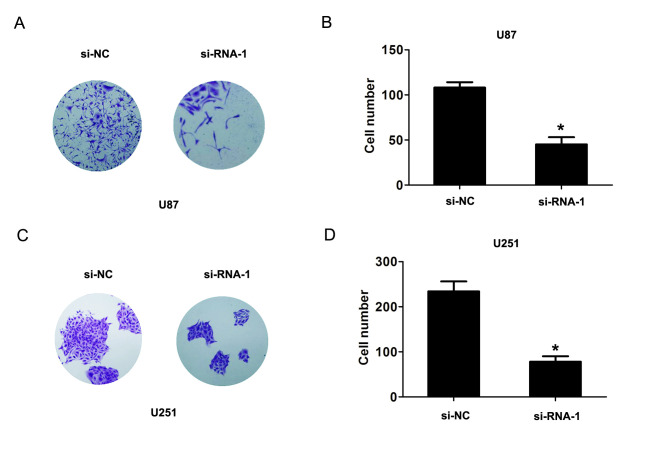
Reduced DUXAP8 expression inhibits cell colony formation ability. The cell colony assays and cell number following transfection of (A) U87 cells and (B) U251 cells with si-NC or si-RNA-1 at 14 days. The cell colony assays and cell number following transfection of (C) U87 cells and (D) U251 cells with si-NC or si-RNA-1 at 14 days. *P<0.05, compared with si-NC. DUXAP8, double homeobox A pseudogene 8; si, small interfering; NC, negative control.

